# Distributed Hypothesis Testing with Privacy Constraints

**DOI:** 10.3390/e21050478

**Published:** 2019-05-07

**Authors:** Atefeh Gilani, Selma Belhadj Amor, Sadaf Salehkalaibar, Vincent Y. F. Tan

**Affiliations:** 1 Department of Electrical and Computer Engineering, College of Engineering, University of Tehran, Tehran 14171614418, Iran; 2 Department of Electrical and Computer Engineering, National University of Singapore, Singapore 117583, Singapore

**Keywords:** hypothesis testing, privacy, mutual information, testing against independence, zero-rate communication

## Abstract

We revisit the distributed hypothesis testing (or hypothesis testing with communication constraints) problem from the viewpoint of privacy. Instead of observing the raw data directly, the transmitter observes a sanitized or randomized version of it. We impose an upper bound on the mutual information between the raw and randomized data. Under this scenario, the receiver, which is also provided with side information, is required to make a decision on whether the null or alternative hypothesis is in effect. We first provide a general lower bound on the type-II exponent for an arbitrary pair of hypotheses. Next, we show that if the distribution under the alternative hypothesis is the product of the marginals of the distribution under the null (i.e., testing against independence), then the exponent is known exactly. Moreover, we show that the strong converse property holds. Using ideas from Euclidean information theory, we also provide an approximate expression for the exponent when the communication rate is low and the privacy level is high. Finally, we illustrate our results with a binary and a Gaussian example.

## 1. Introduction

In the distributed hypothesis testing (or hypothesis testing with communication constraints) problem, some observations from the environment are collected by the sensors in a network. They describe these observations over the network which are finally received by the decision center. The goal is to guess the joint distribution governing the observations at terminals. In particular, there are two possible hypotheses H=0 or H=1, where the joint distribution of the observations is specified under each of them. The performance of this system is characterized by two criteria: the type-I and the type-II error probabilities. The probability of deciding on H=1 (respectively H=0) when the original hypothesis is H=0 (respectively H=1) is referred to as the type-I error (type-II error) probability. There are several approaches for defining the performance of a hypothesis test. First, we can maximize the exponent (exponential rate of decay) of the Bayesian error probability. Second, we can impose that the type-II error probability decays exponentially fast and we can then maximize the exponent of the type-I error probability; this is known as the Hoeffding regime. The approach in this work is the Chernoff-Stein regime in which we upper bound the type-II error probability by a non-vanishing constant and we maximize the exponent of the type-II error probability.

A special case of interest is testing against independence where the joint distribution under H=1 is the product of the marginals under H=0. The optimal exponent of type-II error probability for testing against independence is determined by Ahlswede and Csiszár in [1]. Several extensions of this basic problem are studied for a multi-observer setup [2,3,4,5,6], a multi-decision center setup [7,8] and a setup with security constraints [9]. The main idea of the achievable scheme in these works is typicality testing [10,11]. The sensor finds a jointly typical codeword with its observation and sends the corresponding bin index to the decision center. The final decision is declared based on typicality check of the received codeword with the observation at the center. We note that the coding scheme employed here is reminiscent of those used for source coding with side information [12] and for different variants of the information bottleneck problem [13,14,15,16].

### 1.1. Injecting Privacy Considerations into Our System

We revisit the distributed hypothesis testing problem from a privacy perspective. In many applications such as healthcare systems, there is a need to randomize the data before publishing it. For example, hospitals often have large amounts of medical records of their patients. These records are useful for performing various statistical inference tasks, such as learning about causes of a certain ailment. However, due to privacy considerations of the patients, the data cannot be published as is. The data needs to be sanitized, quantized, perturbed and then be fed to a management center before statistical inference, such as hypothesis testing, is being done.

**Figure 1:**
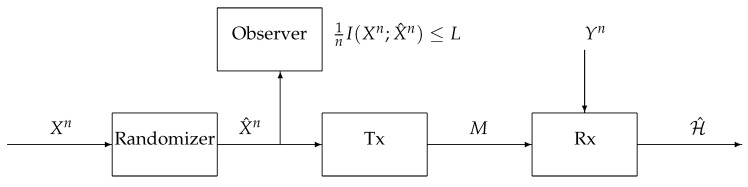
Hypothesis testing with communication and privacy constraints.

In the proposed setup, we use a privacy mechanism to sanitize the observation at the terminal before it is compressed; see Figure 1. The compression is performed at a separate terminal called *transmitter*, which communicates the randomized data over a noiseless link of rate *R* to a receiver. The hypothesis testing is performed using the received data (the compression index and additional side information) to determine the correct hypothesis governing the original observations. The privacy criterion is defined by the mutual information [17,18,19,20] of the published and original data.

There is a long history of research to provide appropriate metrics to measure privacy. To quantify the information leakage an observation $\hat{X}$ can induce on a latent variable *X*, Shannon’s mutual information $I(X;\hat{X})$ is considered in [17,18,19,20]. Smith [18] proposed to use Arimoto’s mutual information of order *∞*, $I_{\infty }\left(X;\hat{X}\right)$. Barthe and Köpf [21,22,23] proposed the maximal information leakage $\max_{P_{X}}I_{\infty }\left(X;\hat{X}\right)$. We refer the reader to [24] for a survey on the existing information leakage measures. A different line of works, in statistics, computer science, and other related fields, concerns *differential privacy*, initially proposed in [25]. Furthermore, a generalized notion—(ϵ,δ)-differential privacy [26]—provides a unified mathematical framework for data privacy. The reader is referred to the survey by Dwork [27] and the statistical framework studied by Wasserman and Zhou [28] and the references therein.

The privacy mechanism can be either memoryless or non-memoryless. In the former, the distribution of the randomized data at each time instant depends on the original sequence at the same time and not on the previous history of the data.

### 1.2. Description of Our System Model

We propose a coding scheme for the proposed setup. The idea is that the sensor, upon observing the source sequence, performs a typicality test and obtains its belief of the hypothesis. If the belief is H=0, it publishes the randomized data based on a specific memoryless mechanism. However, if its belief is H=1, it sends an all-zero sequence to let the transmitter know about its decision. The transmitter communicates the received data, which is a sanitized version of the original data or an all-zero sequence, over the noiseless link to the receiver. In this scheme, the whole privacy mechanism is non-memoryless since the typicality check of the source sequence which uses the history of the observation, determines the published data. It is shown that the achievable error exponent recovers previous results on hypothesis testing with zero and positive communication rates in [10]. Our work is related to a recent work [29] where a general hypothesis testing setup is considered from a privacy perspective. However, in [29], the problem at hand is different from ours. The authors consider equivocation and average distortion as possible measures of privacy whereas we constrain the mutual information between the original and released (published) data.

A difference of the proposed scheme with some previous works is highlighted as follows. The privacy mechanism even if it is memoryless, cannot be viewed as a noiseless link of a rate equivalent to the privacy criterion. Particularly, the proposed model is different from cascade hypothesis testing problem of [8] or similar works [3,4] which consider consecutive noiseless links for data compression and distributed hypothesis testing. The difference comes from the fact that in these works, a codeword is chosen jointly typical with the observed sequence at the terminal and its corresponding index is sent over the noiseless link. However, in our model, the randomized sequence is not necessarily jointly typical with the original sequence. Thus, there is a need for an achievable scheme which lets the transmitter know whether the original data is typical or not.

The problem of hypothesis testing against independence with a memoryless privacy mechanism is also considered. A coding scheme is proposed where the sensor outputs the randomized data based on the memoryless privacy mechanism. The optimality of the achievable type-II error exponent is shown by providing a strong converse. Specializing the optimal error exponent to a binary example shows that an increase in the privacy criterion (a less stringent privacy mechanism) results in a larger type-II error exponent. Thus, there exists a trade-off between privacy and hypothesis testing criteria. The optimal type-II error exponent is further studied for the case of restricted privacy mechanism and zero-rate communication. The Euclidean approach of [30,31,32,33] is used to approximate the error exponent for this regime. The result confirms the trade-off between the privacy criterion and type-II error exponent. Finally, a Gaussian setup is proposed and its optimal error exponent is established.

### 1.3. Main Contributions

The contributions of the paper are listed in the following:

- An achievable type-II error exponent is proposed using a non-memoryless privacy mechanism (Theorem 1 in Section 3);
- The optimal error exponent of testing against independence with a memoryless privacy mechanism is determined. In addition, a strong converse is also proved (Theorem 2 in Section 4.1);
- A binary example is proposed to show the trade-off between the privacy and error exponent (Section 4.3);
- An Euclidean approximation [30] of the error exponent is provided (Section 4.4);
- A Gaussian setup is proposed and its optimal error exponent is derived (Proposition 2 in Section 4.5).

### 1.4. Notation

The notation mostly follows [34]. Random variables are denoted by capital letters, e.g., *X*, *Y*, and their realizations by lower case letters, e.g., *x*, *y*. The alphabet of the random variable *X* is denoted as X. Sequences of random variables and their realizations are denoted by $\left(X_{i},\ldots ,X_{j}\right)$ and $\left(x_{i},\ldots ,x_{j}\right)$ and are abbreviated as $X_{i}^{j}$ and $x_{i}^{j}$. We use the alternative notation $X^{j}$ when i=1. Vectors and matrices are denoted by boldface letters, e.g., k, W. The $\ell_{2}$-norm of k is denoted as ∥k∥. The notation $k^{T}$ denotes the transpose of k.

The probability mass function (pmf) of a discrete random variable *X* is denoted as $P_{X}$, the conditional pmf of *X* given *Y* is denoted as $P_{X|Y}$. The notation $D(P_{X}∥Q_{X})$ denotes the Kullback-Leibler (KL) divergence between two pmfs $P_{X}$ and $Q_{X}$. The total variation distance between two pmfs $P_{X}$ and $Q_{X}$ is denoted by $|P_{X}-Q_{X}|=\frac{1}{2}\sum_{x}\left|P_{X}\left(x\right)-Q_{X}\left(x\right)\right|$. We use $\mathrm{tp}(x^{n},y^{n})$ to denote the joint type of $\left(x^{n},y^{n}\right)$.

For a given $P_{XY}$ and a positive number μ, we denote by $T_{\mu }^{n}\left(P_{XY}\right)$, the set of jointly μ-typical sequences [34], i.e., the set of all $\left(x^{n},y^{n}\right)$ whose joint type is within μ of $P_{XY}$ (in the sense of total-variation distance). The notation $T^{n}\left(P_{X}\right)$ denotes for the type class of the type $P_{X}$.

The notation $h_{b}\left(\cdot \right)$ denotes the binary entropy function, $h_{b}^{-1}\left(\cdot \right)$ its inverse over $\left[0,\frac{1}{2}\right]$, and a⋆b≜a(1−b)+(1−a)b for 0≤a,b≤1. The differential entropy of a continuous random variable *X* is h(X). All logarithms log(·) are taken with respect to base 2.

### 1.5. Organization

The remainder of the paper is organized as follows. Section 2 describes a mathematical setup for our proposed problem. Section 3 discusses hypothesis testing with general distributions. The results for hypothesis testing against independence with a memoryless privacy mechanism are provided in Section 4. The paper is concluded in Section 5.

## 2. System Model

Let X, Y, and $\hat{X}$ be arbitrary finite alphabets and let *n* be a positive integer. Consider the hypothesis testing problem with communication and privacy constraints depicted in Figure 1. The first terminal in the system, the *Randomizer*, receives the sequence $X^{n}=\left(X_{1},\ldots ,X_{n}\right)\in X^{n}$ and outputs the sequence ${\hat{X}}^{n}=\left({\hat{X}}_{1},\ldots ,{\hat{X}}_{n}\right)\in {\hat{X}}^{n}$, which is a noisy version of $X^{n}$ under a *privacy mechanism* determined by the conditional probability distribution $P_{{\hat{X}}^{n}|X^{n}}$; the second terminal, the *Transmitter*, receives the sequence ${\hat{X}}^{n}$; the third terminal, the *Receiver*, observes the side-information sequence $Y^{n}=\left(Y_{1},\ldots ,Y_{n}\right)\in Y^{n}$. Under the null hypothesis

(1) $$\begin{array}{c} H=0:\;\left(X^{n},Y^{n}\right)∼i.i.d.\;P_{XY}, \end{array}$$

whereas under the alternative hypothesis

(2) $$\begin{array}{c} H=1:\;\left(X^{n},Y^{n}\right)∼i.i.d.\;Q_{XY}, \end{array}$$

for two given pmfs $P_{XY}$ and $Q_{XY}$.

The privacy mechanism is described by the conditional pmf $P_{{\hat{X}}^{n}|X^{n}}$ which maps each sequence $X^{n}\in X^{n}$ to a sequence ${\hat{X}}^{n}\in {\hat{X}}^{n}$. For any $\left({\hat{x}}^{n},x^{n},y^{n}\right)\in {\hat{X}}^{n}\times X^{n}\times Y^{n}$, the joint distributions considering the privacy mechanism are given by

(3) $$\begin{array}{cc} \;\;P_{\hat{X}XY}^{n}\left({\hat{x}}^{n},x^{n},y^{n}\right) & \;≜\;P_{{\hat{X}}^{n}|X^{n}}\left({\hat{x}}^{n}|x^{n}\right)\cdot \prod_{i=1}^{n}P_{XY}\left(x_{i},y_{i}\right), \end{array}$$

(4) $$\begin{array}{cc} \;\;Q_{\hat{X}XY}^{n}\left({\hat{x}}^{n},x^{n},y^{n}\right) & \;≜\;P_{{\hat{X}}^{n}|X^{n}}\left({\hat{x}}^{n}|x^{n}\right)\cdot \prod_{i=1}^{n}Q_{XY}\left(x_{i},y_{i}\right). \end{array}$$

A *memoryless/local* privacy mechanism is defined by a conditional pmf $P_{\hat{X}|X}$ which stochastically and independently maps each entry $X_{i}\in X$ of $X^{n}$ to a released ${\hat{X}}_{i}\in \hat{X}$ to construct ${\hat{X}}^{n}$. Consequently, for the memoryless privacy mechanism, the conditional pmf $P_{{\hat{X}}^{n}|X^{n}}\left({\hat{x}}^{n}|x^{n}\right)$ factorizes as follows:

(5) $$\begin{array}{c} P_{{\hat{X}}^{n}|X^{n}}\left({\hat{x}}^{n}|x^{n}\right)=\prod_{i=1}^{n}P_{\hat{X}|X}\left({\hat{x}}_{i}|x_{i}\right)=P_{\hat{X}|X}^{n}\left({\hat{x}}^{n}|x^{n}\right),\;\forall \left({\hat{x}}^{n},x^{n}\right)\in {\hat{X}}^{n}\times X^{n}. \end{array}$$

There is a noise-free bit pipe of rate *R* from the transmitter to the receiver. Upon observing ${\hat{X}}^{n}$, the transmitter computes the message $M=\phi^{\left(n\right)}\left({\hat{X}}^{n}\right)$ using a possibly stochastic encoding function $\phi^{\left(n\right)}:{\hat{X}}^{n}\to \left\{0,\ldots ,\left\lfloor 2^{nR}\right\rfloor \right\}$ and sends it over the bit pipe to the receiver.

The goal of the receiver is to produce a guess of H using a decoding function $g^{\left(n\right)}:Y^{n}\times \left\{0,...,\left\lfloor 2^{nR}\right\rfloor \right\}\to \left\{0,1\right\}$ based on the observation $Y^{n}$ and the received message *M*. Thus the estimate of the hypothesis is $\hat{H}=g^{\left(n\right)}\left(Y^{n},M\right)$.

This induces a partition of the sample space ${\hat{X}}^{n}\times X^{n}\times Y^{n}$ into an acceptance region $A_{n}$ defined as follows:

(6) $$\begin{array}{c} A_{n}≜\left\{\left({\hat{x}}^{n},x^{n},y^{n}\right):g^{\left(n\right)}\left(y^{n},\phi^{\left(n\right)}\left({\hat{x}}^{n}\right)\right)=0\right\}, \end{array}$$

and a rejection region denoted by $A_{n}^{c}$.

**Definition** **1.** *For any ϵ∈[0,1) and for a given rate-privacy pair $\left(R,L\right)\in R_{+}^{2}$, we say that a type-II exponent $\theta \in R_{+}$ is (ϵ,R,L)-achievable if there exists a sequence of functions and conditional pmfs $\left(\phi^{\left(n\right)},g^{\left(n\right)},P_{{\hat{X}}^{n}|X^{n}}\right)$, such that the corresponding sequences of type-I and type-II error probabilities at the receiver are defined as*

(7) $$\begin{array}{c} \alpha_{n}≜P_{\hat{X}XY}^{n}\left(A_{n}^{c}\right)\;and\;\beta_{n}≜Q_{\hat{X}XY}^{n}\left(A_{n}\right), \end{array}$$

*respectively, and they satisfy*

(8) $$\begin{array}{cc} \underset{n\to \infty }{lim sup}\;\alpha_{n} & \leq \epsilon \;\mathrm{and}\;\underset{n\to \infty }{lim inf}\;\frac{1}{n}\log\frac{1}{\beta_{n}}\geq \theta . \end{array}$$

*Furthermore, the privacy measure*

(9) $$T_{n}≜\frac{1}{n}I\left(X^{n};{\hat{X}}^{n}\right),$$

*satisfies*

(10) $$\begin{array}{c} \underset{n\to \infty }{lim sup}\;T_{n}\leq L. \end{array}$$

The optimal exponent $\theta_{\epsilon }^{*}\left(R,L\right)$ is the supremum of all (ϵ,R,L)-achievable $\theta \in R_{+}$.

## 3. General Hypothesis Testing

### 3.1. Achievable Error Exponent

The following presents an achievable error exponent for the proposed setup.

**Theorem** **1.** For a given ϵ∈[0,1) and a rate-privacy pair $\left(R,L\right)\in R_{+}^{2}$, the optimal type-II error exponent $\theta_{\epsilon }^{*}\left(R,L\right)$ for the multiterminal hypothesis testing setup under the privacy constraint L and the rate constraint R satisfies

(11) $$\begin{array}{c} rCl\theta_{\epsilon }^{*}\left(R,L\right)\geq \max_{\begin{array}{c} P_{U|\hat{X}},P_{\hat{X}|X}:\\ R\geq I(U;\hat{X})\\ L\geq I(X;\hat{X}) \end{array}}\;\min_{\begin{array}{c} {\tilde{P}}_{U\hat{X}XY}\in P_{U\hat{X}XY} \end{array}}D\left({\tilde{P}}_{U\hat{X}XY}∥P_{U|\hat{X}}P_{\hat{X}|X}Q_{XY}\;\right), \end{array}$$

*where the set $P_{U\hat{X}XY}$ is defined as*

(12) $$P_{U\hat{X}XY}≜\left\{{\tilde{P}}_{U\hat{X}XY}\;\;\left|\begin{array}{c} {\tilde{P}}_{X}=P_{X},\\ {\tilde{P}}_{UY}=P_{UY},\\ {\tilde{P}}_{U\hat{X}}=P_{U\hat{X}} \end{array}\right.\right\}.$$

*Given $P_{U|\hat{X}}$ and $P_{\hat{X}|X}$, the mutual informations in* (11) *are calculated according to the following joint distribution:*

(13) $$\begin{array}{cc} P_{U\hat{X}|X} & ≜P_{U|\hat{X}}\cdot P_{\hat{X}|X}. \end{array}$$

**Proof.**  The coding scheme is given in the following section. For the analysis, see Appendix A. □

### 3.2. Coding Scheme

In this section, we propose a coding scheme for Theorem 1, under fixed rate and privacy constraints $\left(R,L\right)\in R_{+}^{2}$. Fix the joint distribution $P_{U\hat{X}XY}$ as in (13). Let $P_{U}\left(u\right)$ be the marginal distribution of U∈U defined as

(14) $$\begin{array}{c} P_{U}\left(u\right)≜\sum_{\hat{x}\in \hat{X}}P_{U|\hat{X}}\left(u|\hat{x}\right)\sum_{x\in X}P_{\hat{X}X}\left(\hat{x},x\right). \end{array}$$

Fix positive μ>0 and ζ>0, an arbitrary blocklength *n* and two conditional pmfs $P_{\hat{X}|X}$ and $P_{U|\hat{X}}$ over finite auxiliary alphabets $\hat{X}$ and U. Fix also the rate and privacy leakage level as

(15) $$\begin{array}{c} R=I\left(U;\hat{X}\right)+\mu ,\;\mathrm{and}\;L=I\left(\hat{X};X\right)+\zeta . \end{array}$$

*Codebook Generation*: Randomly and independently generate a codebook

(16) $$\begin{array}{c} C_{U}≜\left\{U^{n}\left(m\right):m\in \left\{0,\ldots ,\left\lfloor 2^{nR}\right\rfloor \right\}\right\}, \end{array}$$

by drawing $U^{n}\left(m\right)$ in an i.i.d. manner according to $P_{U}$. The codebook is revealed to all terminals.

*Randomizer*: Upon observing $x^{n}$, it checks whether $x^{n}\in T_{\mu /4}^{n}\left(P_{X}\right).$ If successful, it outputs the sequence ${\hat{x}}^{n}$ where its *i*-th component ${\hat{x}}_{i}$ is generated based on $x_{i}$, according to $P_{\hat{X}|X}\left({\hat{x}}_{i}|x_{i}\right)$. If the typicality check is not successful, the randomizer then outputs $0^{n}$ which is an all-zero sequence of length *n*, where ${\hat{x}}^{n}=0^{n}$.

*Transmitter*: Upon observing ${\hat{x}}^{n}$, if ${\hat{x}}^{n}\neq 0^{n}$, the transmitter finds an index *m* such that $\left(u^{n}\left(m\right),{\hat{x}}^{n}\right)\in T_{\mu /2}^{n}\left(P_{U\hat{X}}\right).$ If successful, it sends the index *m* over the noiseless link to the receiver. Otherwise, if the typicality check is not successful or ${\hat{x}}^{n}=0^{n}$, it sends m=0.

*Receiver*: Upon observing $y^{n}$ and receiving the index *m*, if m=0, the receiver declares $\hat{H}=1$. If m≠0, it checks whether $\left(u^{n}\left(m\right),y^{n}\right)\in T_{\mu }^{n}\left(P_{UY}\right).$ If the test is successful, the receiver declares $\hat{H}=0$; otherwise, it sets $\hat{H}=1$.

**Remark** **1.** *In the above scheme, the sequence ${\hat{X}}^{n}$ is chosen to be an n-length zero-sequence when the randomizer finds that $X^{n}$ is not typical according to $P_{X}$. Thus, the privacy mechanism is not memoryless and the sequence ${\hat{X}}^{n}$ is not identically and independently distributed (i.i.d.). A detailed analysis in Appendix A shows that the privacy criterion is not larger than L as the blocklength n→∞.*

### 3.3. Discussion

In the following, we discuss some special cases. First, suppose that R=0. The following corollary shows that Theorem 1 recovers Han’s result [1] for distributed hypothesis testing with zero-rate communication.

**Corollary** **1** (Theorem 5 in [10])**.** *Suppose that $Q_{XY}>0$. For all ϵ∈[0,1), the optimal error exponent of the zero-rate communication for any privacy mechanism (including non-memoryless mechanisms) is given by the following:*

(17) $$\begin{array}{c} \theta_{\epsilon }^{*}\left(0,L\right)=\min_{\begin{array}{c} {\tilde{P}}_{XY}:\\ {\tilde{P}}_{X}=P_{X}\\ {\tilde{P}}_{Y}=P_{Y} \end{array}}D\left({\tilde{P}}_{XY}∥Q_{XY}\;\right). \end{array}$$

**Proof.**  The proof of achievability follows by Theorem 1, in which $\hat{X}$ is arbitrary and the auxiliary U=∅ due to the zero-rate constraint. The proof of the strong converse follows along the same lines as [35]. □

**Remark** **2.** *Consider the case of R>0 and L=0 where $\hat{X}$ is independent of X. Using Theorem 1, the optimal error exponent is lower bounded as follows:*

(18) $$\begin{array}{c} \theta_{\epsilon }^{*}\left(R,0\right)\geq \min_{\begin{array}{c} {\tilde{P}}_{XY}:\\ {\tilde{P}}_{X}=P_{X}\\ {\tilde{P}}_{Y}=P_{Y} \end{array}}D\left({\tilde{P}}_{XY}∥Q_{XY}\;\right). \end{array}$$

*However, there is no known converse result in this case where the communication rate is positive. Comparing this special case with the one in Corollary 1 shows that the proposed model does not, in general, admit symmetry between the rate and privacy constraints. However, we will see from some specific examples in the following that the roles of R and L are symmetric.*

Now, suppose that *L* is so large such that L>H(X). The following corollary shows that Theorem 1 recovers Han’s result in [10] for distributed hypothesis testing over a rate-*R* communication link.

**Corollary** **2** (Theorem 2 in [10])**.** *Assuming L>H(X), the optimal error exponent is lower bounded as the following:*

(19) $$\theta_{\epsilon }^{*}\left(R,L\right)\geq \max_{\begin{array}{c} P_{U|X}:\\ R\geq I(U;X) \end{array}}\;\;\;\min_{\begin{array}{c} {\tilde{P}}_{UXY}:\\ {\tilde{P}}_{UX}=P_{UX}\\ {\tilde{P}}_{UY}=P_{UY} \end{array}}D\left({\tilde{P}}_{UXY}∥P_{U|X}Q_{XY}\right).$$

**Proof.**  The proof follows from Theorem 1 by specializing to $\hat{X}=X$. □

The above two special cases reveal a trade-off between the privacy criterion and the achievable error exponent when the communication rate is positive, i.e., R>0. An increase in *L* results in a larger achievable error exponent. This observation is further illustrated by an example in Section 4.3 to follow.

## 4. Hypothesis Testing against Independence with a Memoryless Privacy Mechanism

In this section, we consider testing against independence where the joint pmf under H=1 factorizes as follows:

(20) $$\begin{array}{c} Q_{XY}=P_{X}\cdot P_{Y}. \end{array}$$

The privacy mechanism is assumed to be memoryless here.

### 4.1. Optimal Error Exponent

The following theorem, which includes a strong converse, states the optimal error exponent for this special case.

**Theorem** **2.** *For any $\left(R,L\right)\in R_{+}^{2}$, define*

(21) $$\theta_{\epsilon }^{*}\left(R,L\right)=\;\;\max_{\begin{array}{c} P_{U|\hat{X}},P_{\hat{X}|X}:\\ \;R\geq I(U;\hat{X})\\ \;L\geq I(X;\hat{X}) \end{array}}\;\;I\left(U;Y\right).$$

*Then, for any ϵ∈[0,1) and any $\left(R,L\right)\in R_{+}^{2}$, the optimal error exponent for testing against independence when using a memoryless privacy mechanism is given by* (21)*, where it suffices to choose $\left|U|\leq \right|\hat{X}|+1$ and $|\hat{X}\left|\leq |X\right|$ according to Caratheodory’s theorem [36] (Theorem 15.3.5).*

**Proof.**  The coding scheme is given in the following section. For the rest of proof, see Appendix B. □

### 4.2. Coding Scheme

In this section, we propose a coding scheme for Theorem 2. Fix the joint distribution as in (13), and the rate and privacy constraints as in (15). Generate the codebook $C_{U}$ as in (16).

*Randomizer*: Upon observing $x^{n}$, it outputs the sequence ${\hat{x}}^{n}$ in which the *i*-th component ${\hat{x}}_{i}$ is generated based on $x_{i}$, according to $P_{\hat{X}|X}\left({\hat{x}}_{i}|x_{i}\right)$.

*Transmitter*: It finds an index *m* such that $\left(u^{n}\left(m\right),{\hat{x}}^{n}\right)\in T_{\mu /2}^{n}\left(P_{U\hat{X}}\right).$ If successful, it sends the index *m* over the noiseless link to the receiver. Otherwise, it sends m=0.

*Receiver*: Upon observing $y^{n}$ and receiving the index *m*, if m=0, the receiver declares $\hat{H}=1$. If m≠0, it checks whether $\left(u^{n}\left(m\right),y^{n}\right)\in T_{\mu }^{n}\left(P_{UY}\right).$ If the test is successful, the receiver declares $\hat{H}=0$; otherwise, it sets $\hat{H}=1$.

**Remark** **3.** *In the above scheme, the sequence ${\hat{X}}^{n}$ is i.i.d. since it is generated based on the memoryless mechanism $P_{\hat{X}|X}$.*

When the communication rate is positive, there exists a trade-off between the optimal error exponent and the privacy criterion. The following example elucidates this trade-off.

### 4.3. Binary Example

In this section, we study hypothesis testing against independence for a binary example. Suppose that under both hypotheses, we have $X∼\mathrm{Bern}(\frac{1}{2})$. Under the null hypothesis,

(22) $$\begin{array}{c} H=0:\;Y=X⊕N,\;N∼\mathrm{Bern}(q) \end{array}$$

for some 0≤q≤1, where *N* is independent of *X*. Under the alternative hypothesis

(23) $$H=1:\;Y∼\mathrm{Bern}\left(\frac{1}{2}\right),$$

where *Y* is independent of *X*. The cardinality constraint shows that it suffices to choose $|\hat{X}|=2$. Among all possible privacy mechanisms, the choice of $P_{\hat{X}|X}\left(1|0\right)=P_{\hat{X}|X}\left(1|1\right)$ and $P_{\hat{X}|X}\left(0|0\right)=P_{\hat{X}|X}\left(0|1\right)$ minimizes the mutual information $I(X;\hat{X})$. Thus, we restrict to this choice which also results in $\hat{X}∼\mathrm{Bern}\left(\frac{1}{2}\right)$.

The cardinality bound on the auxiliary random variable *U* is |U|≤3. The following proposition states that it is also optimal to choose $P_{U|\hat{X}}$ to be a BSC.

**Proposition** **1.** *The optimal error exponent of the proposed binary setup is given by the following:*

(24) $$\begin{array}{cc} \theta_{\epsilon }^{*}\left(R,L\right) & =1-h_{b}\left(q⋆h_{b}^{-1}\left(1-L\right)⋆h_{b}^{-1}\left(1-R\right)\right). \end{array}$$

**Proof.**  For the proof of achievability, choose the following auxiliary random variables:

(25) $$\begin{array}{cc} \hat{X} & =X⊕\hat{Z},\;\hat{Z}∼\mathrm{Bern}\left(p_{1}\right) \end{array}$$

(26) $$\begin{array}{cc} U & =\hat{X}⊕Z,\;Z∼\mathrm{Bern}\left(p_{2}\right), \end{array}$$

for some $0\leq p_{1},p_{2}\leq 1$ where $\hat{Z}$ and *Z* are independent of *X* and $\left(X,\hat{X}\right)$, respectively. The optimal error exponent of Theorem 2 reduces to the following:

(27) $$\begin{array}{cc} \theta_{\epsilon }^{*}\left(R,L\right) & =\max_{\begin{array}{c} 0\leq p_{1},p_{2}\leq 1:\\ \;R\geq 1-h_{b}\left(p_{2}\right)\;\\ L\geq 1-h_{b}\left(p_{1}\right) \end{array}}1-h_{b}\left(q⋆p_{1}⋆p_{2}\right), \end{array}$$

which can be simplified to (24). For the proof of the converse, see Appendix C. □

**Figure 2:**
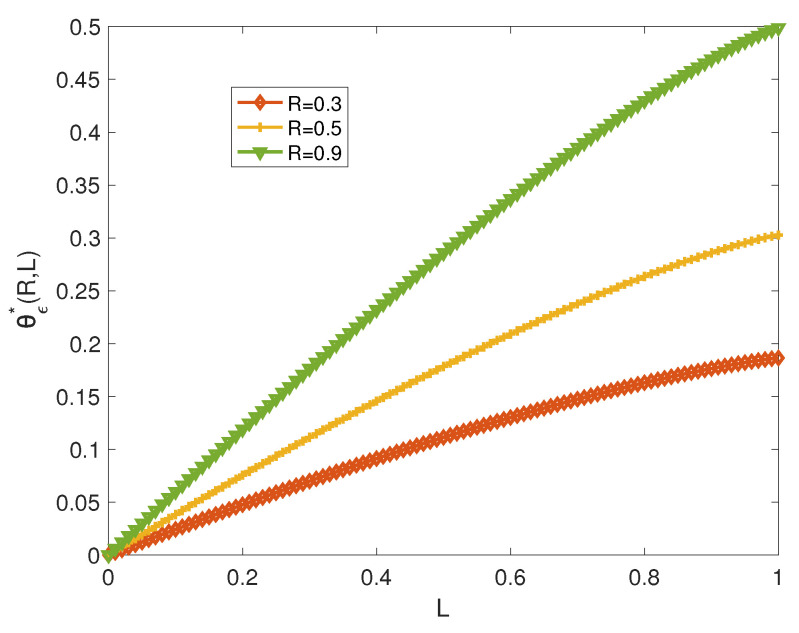
$\theta_{\epsilon }^{*}\left(R,L\right)$ versus *L* for q=0.1 and various values of *R*.

Figure 2 illustrates the error exponent versus the privacy parameter *L* for a fixed rate *R*. There is clearly a trade-off between $\theta_{\epsilon }^{*}\left(R,L\right)$ and *L*. For a less stringent privacy requirement (large *L*), the error exponent $\theta_{\epsilon }^{*}\left(R,L\right)$ increases.

### 4.4. Euclidean Approximation

In this section, we propose Euclidean approximations [30,31] for the optimal error exponent of testing against independence scenario (Theorem 2) when R≈0 and L≈0. Consider the optimal error exponent as follows:

(28) $$\begin{array}{c} \theta_{\epsilon }^{*}\left(R,L\right)=\max_{\begin{array}{c} P_{U|\hat{X}},P_{\hat{X}|X}:\\ \;R\geq I(U;\hat{X})\;\\ L\geq I(X;\hat{X}) \end{array}}\;\;I\left(U;Y\right). \end{array}$$

Let W of dimension |Y|×|X|, denote the transition matrix $P_{Y|X}$, which is itself induced by $P_{X}$ and the joint distribution $P_{XY}$. Now, consider the rate constraint as follows:

(29) $$\begin{array}{cc} I(U;\hat{X}) & =\sum_{u\in U}P_{U}\left(u\right)D\left(P_{\hat{X}|U}\left(\cdot |u\right)∥P_{\hat{X}}\right)\leq R. \end{array}$$

Assuming R≈0, we let $P_{\hat{X}|U}\left(\cdot |u\right)$ be a local perturbation from $P_{\hat{X}}\left(\cdot \right)$, where we have

(30) $$P_{\hat{X}|U}\left(\cdot |u\right)=P_{\hat{X}}\left(\cdot \right)+\psi_{u}\left(\cdot \right),$$

for a perturbation $\psi_{u}\left(\cdot \right)$ satisfying

(31) $$\begin{array}{c} \sum_{\hat{x}\in \hat{X}}\psi_{u}\left(\hat{x}\right)=0, \end{array}$$

in order to preserve the row stochasticity of $P_{\hat{X}|U}$. Using a $\chi^{2}$-approximation [30], we can write:

(32) $$\begin{array}{cc} D\left(P_{\hat{X}|U}\left(\cdot |u\right)∥P_{\hat{X}}\right) & \approx \frac{1}{2}\cdot \log e\cdot {∥v_{u}∥}^{2}, \end{array}$$

where $v_{u}$ denotes the length-$|\hat{X}|$ column vector of weighted perturbations whose $\hat{x}$-th component is defined as:

(33) $$\begin{array}{cc} v_{u}\left(\hat{x}\right) & ≜\frac{1}{\sqrt{P_{\hat{X}}\left(\hat{x}\right)}}\cdot \psi_{u}\left(\hat{x}\right),\;\forall \hat{x}\in \hat{X}. \end{array}$$

Using the above definition, the rate constraint in (29) can be written as:

(34) $$\begin{array}{cc} \sum_{u\in U}P_{U}\left(u\right){∥v_{u}∥}^{2} & \leq \frac{2R}{\log e}. \end{array}$$

Similarly, consider the privacy constraint as the following:

(35) $$\begin{array}{cc} I(X;\hat{X}) & =\sum_{\hat{x}\in \hat{X}}P_{\hat{X}}\left(\hat{x}\right)D\left(P_{X|\hat{X}}\left(\cdot |\hat{x}\right)∥P_{X}\right)\leq L. \end{array}$$

Assuming L≈0, we let $P_{X|\hat{X}}\left(\cdot |\hat{x}\right)$ be a local perturbation from $P_{X}\left(\cdot \right)$ where

(36) $$\begin{array}{c} P_{X|\hat{X}}\left(\cdot |\hat{x}\right)=P_{X}\left(\cdot \right)+\phi_{\hat{x}}\left(\cdot \right), \end{array}$$

for a perturbation $\phi_{\hat{x}}\left(\cdot \right)$ that satifies:

(37) $$\begin{array}{c} \sum_{x\in X}\phi_{\hat{x}}\left(x\right)=0. \end{array}$$

Again, using a $\chi^{2}$-approximation, we obtain the following:

(38) $$\begin{array}{c} D\left(P_{X|\hat{X}}\left(\cdot |\hat{x}\right)∥P_{X}\right)\approx \frac{1}{2}\;\log e\;{∥v_{\hat{x}}∥}^{2}, \end{array}$$

where $v_{\hat{x}}$ is a length-|X| column vector and its *x*-th component is defined as follows:

(39) $$\begin{array}{cc} v_{\hat{x}}\left(x\right) & ≜\frac{1}{\sqrt{P_{X}\left(x\right)}}\cdot \phi_{\hat{x}}\left(x\right),\;\forall x\in X. \end{array}$$

Thus, the privacy constraint in (35) can be written as:

(40) $$\begin{array}{cc} \sum_{\hat{x}\in \hat{X}}P_{\hat{X}}\left(\hat{x}\right){∥v_{\hat{x}}∥}^{2} & \leq \frac{2L}{\log e}. \end{array}$$

For any x∈X and u∈U, we define the following:

(41)Λu(x)≜∑x^∈X^ψu(x^)ϕx^(x)(42)=PX(x)∑x^∈X^PX^(x^)vu(x^)vx^(x),

and the corresponding length-|X| column vector $\Lambda_{u}$ defined as follows:

(43) $$\begin{array}{c} \Lambda_{u}=\left[\sqrt{P_{X}}\right]V_{\hat{X}}\left[\sqrt{P_{\hat{X}}}\right]v_{u}, \end{array}$$

where $\left[\sqrt{P_{X}}\right]$ denotes a diagonal |X|×|X|-matrix, so that its (x,x)-th element (x∈X) is $\sqrt{P_{X}\left(x\right)}$, and $\left[\sqrt{P_{\hat{X}}}\right]$ is defined similarly. Moreover, $V_{\hat{X}}$ refers to the $\left|X|\times \right|\hat{X}|$-matrix defined as follows:

(44) $$\begin{array}{c} V_{\hat{X}}≜\left[\begin{array}{cccccc} v_{1} & v_{2} & \ldots & v_{\hat{x}} & \ldots & v_{|\hat{X}|} \end{array}\right]. \end{array}$$

Let ${\left[\sqrt{P_{Y}}\right]}^{-1}$ be the inverse of diagonal |Y|×|Y|-matrix $\left[\sqrt{P_{Y}}\right]$. As shown in Appendix D, the optimization problem in (28) can be written as follows:

(45) $$\begin{array}{c} \max_{\begin{array}{c} {\left\{v_{u}\right\}}_{u\in U},V_{\hat{X}}:\\ -\sqrt{P_{\hat{X}}\left(\hat{x}\right)}\leq v_{u}\left(\hat{x}\right)\leq \frac{1-P_{\hat{X}}\left(\hat{x}\right)}{\sqrt{P_{\hat{X}}\left(\hat{x}\right)}}\\ -\sqrt{P_{X}\left(x\right)}\leq v_{\hat{x}}\left(x\right)\leq \frac{1-P_{X}\left(x\right)}{\sqrt{P_{X}\left(x\right)}} \end{array}}\;\frac{1}{2}\;\log e\;\left[\sum_{u\in U}P_{U}\left(u\right)\cdot {∥{\left[\sqrt{P_{Y}}\right]}^{-1}W\left[\sqrt{P_{X}}\right]V_{\hat{X}}\left[\sqrt{P_{\hat{X}}}\right]V_{u}∥}^{2}\right] \end{array}$$

(46) $$\begin{array}{c} \mathrm{subject}\;\mathrm{to}:\;\sum_{u\in U}P_{U}\left(u\right){∥v_{u}∥}^{2}\leq \frac{2R}{\log e}, \end{array}$$

(47) $$\begin{array}{c} \;\sum_{\hat{x}\in \hat{X}}P_{\hat{X}}\left(\hat{x}\right){∥v_{\hat{x}}∥}^{2}\leq \frac{2L}{\log e}. \end{array}$$

The following example specializes the above approximation to the binary case.

**Example** **1.** *Consider the binary setup of Example 4.3 and the choice of auxiliary random variables in* (26). *Since the privacy mechanism is assumed to be a BSC, we have*

(48) $$\begin{array}{c} P_{X}={\left[\frac{1}{2}\;\;\;\frac{1}{2}\right]}^{T},\;P_{\hat{X}}={\left[\frac{1}{2}\;\;\;\frac{1}{2}\right]}^{T}, \end{array}$$

*Now, we consider the vectors $v_{u=0}$ and $v_{u=1}$ defined as*

(49) $$\begin{array}{cc} v_{u=0} & ={\left[\begin{array}{cc} \sqrt{2}\xi_{1} & -\sqrt{2}\xi_{1} \end{array}\right]}^{T}, \end{array}$$

(50) $$\begin{array}{cc} v_{u=1} & ={\left[\begin{array}{cc} -\sqrt{2}\xi_{1} & \sqrt{2}\xi_{1} \end{array}\right]}^{T}. \end{array}$$

*for some positive $\xi_{1}$. This yields the following:*

(51) $$\begin{array}{cc} P_{\hat{X}|U=0} & =P_{\hat{X}}+{\left[\xi_{1}\;\;-\xi_{1}\right]}^{T}, \end{array}$$

(52) $$\begin{array}{cc} P_{\hat{X}|U=1} & =P_{\hat{X}}+{\left[-\xi_{1}\;\;\;\xi_{1}\right]}^{T} \end{array}$$

*We also choose the vectors $v_{\hat{x}=0}$ and $v_{\hat{x}=1}$ as follows:*

(53) $$\begin{array}{cc} v_{\hat{x}=0} & ={\left[\begin{array}{cc} \sqrt{2}\xi_{2} & -\sqrt{2}\xi_{2} \end{array}\right]}^{T}, \end{array}$$

(54) $$\begin{array}{cc} v_{\hat{x}=1} & ={\left[\begin{array}{cc} -\sqrt{2}\xi_{2} & \sqrt{2}\xi_{2} \end{array}\right]}^{T}, \end{array}$$

*which results in*

(55) $$\begin{array}{cc} P_{X|\hat{X}=0} & =P_{X}+{\left[\xi_{2}\;\;-\xi_{2}\right]}^{T}, \end{array}$$

(56) $$\begin{array}{cc} P_{X|\hat{X}=1} & =P_{X}+{\left[-\xi_{2}\;\;\;\xi_{2}\right]}^{T}. \end{array}$$

*Notice that the matrix W is given by*

(57) $$\begin{array}{c} W=\left[\begin{array}{cc} 1-q & q\\ q & 1-q \end{array}\right]. \end{array}$$

*Thus, the optimization problem in* (45) *and* (47) *reduces to the following:*

(58) $$\begin{array}{c} \max_{-\frac{1}{2}\leq \xi_{1},\xi_{2}\leq \frac{1}{2}}\;8\;\log e\;{\left(1-2q\right)}^{2}\;|\xi_{1}{|}^{2}\;{\left|\xi_{2}\right|}^{2} \end{array}$$

(59) $$\begin{array}{c} \;subject\;to:\;4\;|\xi_{1}{|}^{2}\leq \frac{2R}{\log e}\;and\;4\;{\left|\xi_{2}\right|}^{2}\leq \frac{2L}{\log e}. \end{array}$$

*Solving the above optimization yields*

(60) $$\begin{array}{cc} \theta_{\epsilon }^{*}\left(R\approx 0,L\approx 0\right) & \approx \frac{2}{\log e}\;{\left(1-2q\right)}^{2}\;R\;L. \end{array}$$

**Figure 3:**
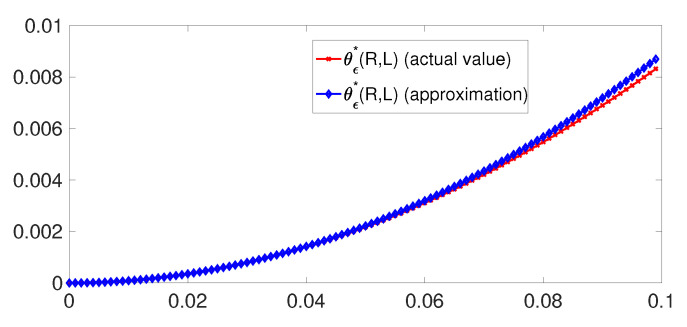
$\theta_{\epsilon }^{*}\left(R\approx 0,L\approx 0\right)$ versus *L* for q=0.1 and R=L.

*For some values of parameters, the approximation in* (60) *is compared to the error exponent of* (24) *in Figure 3. We observe that when R=L≈0, the approximation turns out to be excellent.*

**Remark** **4.** *The trade-off between the optimal error exponent and the privacy can again be verified from* (60) *in the case of L≈0 and R≈0. As L becomes larger (which corresponds to a less stringent privacy requirement), the error exponent also increases. For a fixed error exponent, a trade-off between R and L exists. An increase in R results in a decrease of L.*

### 4.5. Gaussian Setup

In this section, we consider hypothesis testing against independence over a Gaussian example. Suppose that X∼N(0,1) and under the null hypothesis H=0, the sources *X* and *Y* are jointly Gaussian random variables distributed as $N(0,G_{XY})$, where $G_{XY}$ is defined as the following:

(61) $$\begin{array}{c} G_{XY}\overset{\Delta }{=}\left[\begin{array}{cc} 1 & \rho \\ \rho & 1 \end{array}\right], \end{array}$$

for some 0≤ρ≤1.

Under the alternative hypothesis H=1, we assume that *X* and *Y* are independent Gaussian random variables, each distributed as N(0,1). Consider the privacy constraint as follows:

(62) $$\begin{array}{cc} L & \geq I\left(X;\hat{X}\right)=h\left(X\right)-h\left(X|\hat{X}\right). \end{array}$$

For a Gaussian source *X*, the conditional entropy $h(X|\hat{X})$ is maximized for a jointly Gaussian $\left(X,\hat{X}\right)$. This choice minimizes the RHS of (62). Thus, without loss of optimality, we choose

(63) $$\begin{array}{c} X=\hat{X}+Z,\;Z∼N\left(0,2^{-2L}\right), \end{array}$$

where *Z* is independent of $\hat{X}$. The following proposition states that it is optimal to choose *U* jointly Gaussian with $\left(X,\hat{X},Y\right)$.

**Proposition** **2.** *The optimal error exponent of the proposed Gaussian setup is given by*

(64) $$\begin{array}{c} \;\theta_{\epsilon }^{*}\left(R,L\right)\;=\;\frac{1}{2}\log\left(\frac{1}{1-\rho^{2}\cdot \left(1-2^{-2R}\right)\cdot \left(1-2^{-2L}\right)}\right). \end{array}$$

**Proof.**  For the proof of achievability, we choose $\hat{X}$ as in (63). Also, let

(65) $$\begin{array}{c} \hat{X}=U+\hat{Z},\;\hat{Z}∼N\left(0,\beta^{2}\right), \end{array}$$

for some $\beta^{2}\geq 0$, where $\hat{Z}$ is independent of *U*. It can be shown that Theorem 2 remains valid when it is extended to the continuous alphabet [5]. For the details of the simplification and also the proof of converse, see Appendix E. □

**Remark** **5.** *If L=∞, the above proposition recovers the optimal error exponent of Rahman and Wagner [5] (Corollary 7) for testing against independence of Gaussian sources over a noiseless link of rate R.*

## 5. Summary and Discussion

In this paper, distributed hypothesis testing with privacy constraints is considered. A coding scheme is proposed where the sensor decides on one of hypotheses and generates the randomized data based on its decision. The transmitter describes the randomized data over a noiseless link to the receiver. The privacy mechanism in this scheme is non-memoryless. The special case of testing against independence with a memoryless privacy mechanism is studied in detail. The optimal type-II error exponent of this case is established, together with a strong converse. A binary example is proposed where the trade-off between the privacy criterion and the error exponent is reported. Euclidean approximations are provided for the case in which the privacy level is high and and the communication rate is vanishingly small. The optimal type-II error exponent of a Gaussian setup is also established.

A future line of research is to study the second-order asymptotics of our model. The second-order analysis of distributed hypothesis testing without privacy constraints and with zero-rate communication was studied in [37]. In our proposed model, the trade-off between the privacy and type-II error exponent is observed, i.e., a less stringent privacy requirement yields a larger error exponent. The next step is to see whether the trade-off between privacy and error exponent affects the second-order term.

Another potential line for future research is to consider other metrics of privacy instead of the mutual information. A possible candidate is to use the maximal leakage [21,22,23] and to analyze the performance in tandem with distributed hypothesis testing problem.

## Appendix A. Proof of Theorem 1

The analysis is based on the scheme described in Section 3.2.

*Error Probability Analysis:* We analyze the type-I and type-II error probabilities averaged over all random codebooks. By standard arguments [36] (p. 204), it can be shown that there exists at least one codebook that satisfies constraints on the error probabilities.

For the considered μ>0 and the considered blocklength *n*, let $P_{\mu }^{n}$ be the set of all joint types $\pi_{U\hat{X}XY}$ over $U^{n}\times {\hat{X}}^{n}\times X^{n}\times Y^{n}$ which satisfy the following constraints:

(A1) $$\begin{array}{cc} \left|\pi_{X}-P_{X}\right| & \leq \mu /4, \end{array}$$

(A2) $$\begin{array}{cc} \left|\pi_{U\hat{X}}-P_{U\hat{X}}\right| & \leq \mu /2, \end{array}$$

(A3) $$\begin{array}{cc} \left|\pi_{UY}-P_{UY}\right| & \leq \mu . \end{array}$$

First, we analyze the type-I error probability. For the case of M≠0, we define the following event:

(A4) $$\begin{array}{c} E≜\left\{\left(U^{n}\left(M\right),Y^{n}\right)\notin T_{\mu }^{n}\left(P_{UY}\right)\right\}. \end{array}$$

Thus, type-I error probability can be upper bounded as follows:

(A5)αn≤PrX^n=0norM=0orE|H=0(A6)≤PrX^n=0n|H=0+PrM=0|X^n≠0n,H=0+PrE|M≠0,X^n≠0n,H=0(A7)≤ϵ/3+PrM=0|X^n≠0n,H=0+PrE|M≠0,X^n≠0n,H=0(A8)≤ϵ/3+ϵ/3+PrE|M≠0,X^n≠0n,H=0(A9)≤ϵ/3+ϵ/3+ϵ/3(A10)=ϵ,

where (A7) follows from AEP [36] (Theorem 3.1.1); (A8) follows from the covering lemma [34] (Lemma 3.3) and the rate constraint (15), (A10) follows from Markov lemma [34] (Lemma 12.1). In all justifications, *n* is taken to be sufficiently large.

Next, we analyze the type-II error probability. The acceptance region at the receiver is

(A11) $$\begin{array}{c} A_{n}^{\mathrm{Rx}}=\underset{m}{⋃}\left\{\left({\hat{x}}^{n},x^{n},y^{n}\right):{\hat{x}}^{n}\neq 0^{n},\left(u^{n}\left(m\right),{\hat{x}}^{n},x^{n},y^{n}\right)\in T_{\mu }^{n}\left(P_{U\hat{X}XY}\right)\right\}. \end{array}$$

The set $A_{n}^{\mathrm{Rx}}$ is contained within the following acceptance region ${\bar{A}}_{n}$:

(A12) $$\begin{array}{c} {\bar{A}}_{n}=\underset{m}{⋃}\left\{\left({\hat{x}}^{n},x^{n},y^{n}\right):{\hat{x}}^{n}\neq 0^{n},\left(u^{n}\left(m\right),{\hat{x}}^{n},x^{n},y^{n}\right)\in \underset{\pi \in P_{\mu }^{n}}{⋃}T^{n}\left(\pi \right)\right\}. \end{array}$$

Let $F_{m}≜\left\{\left(U^{n}\left(m\right),{\hat{X}}^{n},X^{n},Y^{n}\right)\;\in \;P_{\mu }^{n}\right\}$. Therefore, the average of type-II error probability over all codebooks is upper bounded as follows:

(A13)EC[βn]≤QX^XYnA¯n(A14)≤∑mPrX^n≠0n,Fm|H=1(A15)≤∑mPrFm|X^n≠0n,H=1(A16)≤2nR·(n+1)|U|·|X^|·|X|·|Y|·maxπUX^XY∈Pμn2−nD(πUX^XY∥PUPX^|XQXY)(A17)=(n+1)|U|·|X^|·|X|·|Y|·2−nθ˜μ,

where

(A18) $${\tilde{\theta }}_{\mu }≜\min_{\pi_{U\hat{X}XY}\in P_{\mu }^{n}}D\left(\pi_{U\hat{X}XY}∥P_{U}P_{\hat{X}|X}Q_{XY}\right)-R,$$

and (A16) follows from the upper bound of Sanov’s theorem [36] (Theorem 11.4.1). Hence,

(A19)θ˜μ=minπUX^XY∈PμnD(πUX^XY∥PUPX^|XQXY)−R(A20)=minπUX^XY∈PμnD(πUX^XY∥PUPX^|XQXY)−I(U;X^)−μ(A21)=minπUX^XY∈PμnD(πUX^XY∥PU|X^PX^|XQXY)+δ(μ),

where δ(μ)→0 as μ→0. Equality (A20) follows from the rate constraint in (15) and (A21) holds because $|\pi_{U\hat{X}}-P_{U\hat{X}}|<\mu /2$.

*Privacy Analysis*: We first analyze the privacy under H=0. Notice that ${\hat{X}}^{n}$ is not necessarily i.i.d. because according to the scheme in Section 3.2, ${\hat{X}}^{n}$ is forced to be an all-zero sequence if the Randomizer decides that $X^{n}$ is not typical. However, conditioned on the event that $X^{n}\in T_{\mu }^{n}\left(P_{X}\right)$, the sequence ${\hat{X}}^{n}$ is i.i.d. according to the conditional pmf $P_{\hat{X}|X}$. The privacy measure $T_{n}$ satisfies

(A22) $$\begin{array}{c} nT_{n}=I\left(X^{n};{\hat{X}}^{n}\right)=H\left({\hat{X}}^{n}\right)-H\left({\hat{X}}^{n}|X^{n}\right). \end{array}$$

We now provide a lower bound on $H({\hat{X}}^{n}|X^{n})$ as follows

(A23) $$\begin{array}{cc} H({\hat{X}}^{n}|X^{n}) & \geq \;\;\sum_{x^{n}\in T_{\mu }^{n}\left(P_{X}\right)}\;\;P_{X}^{n}\left(x^{n}\right)H\left({\hat{X}}^{n}|X^{n}=x^{n}\right) \end{array}$$

For any $x^{n}\in T_{\mu }^{n}\left(P_{X}\right)$ and for $\mu^{\prime }>\mu$, it holds that

(A24)H(X^n|Xn=xn)=−∑x^n∈X^nPX^|Xn(x^n|xn)logPX^|Xn(x^n|xn)(A25)≥−∑x^n∈Tμ′n(PX^|X(·|xn))PX^|Xn(x^n|xn)logPX^|Xn(x^n|xn)(A26)≥−∑x^n∈Tμ′n(PX^|X(·|xn))PX^|Xn(x^n|xn)log2−n(1−μ′)H(X^|X)(A27)≥n(1−μ′)2H(X^|X)

where (A26) is true because for any ${\hat{x}}^{n}\in T_{\mu^{\prime }}^{n}\left(P_{\hat{X}|X}\left(\cdot |x^{n}\right)\right)$, it holds that $P_{\hat{X}|X}^{n}\left({\hat{x}}^{n}|x^{n}\right)\leq 2^{-n\left(1-\mu^{\prime }\right)H\left(\hat{X}|X\right)}$, and (A27) follows because the conditional typicality lemma [34] (Chapter 2) implies that $P_{\hat{X}|X}^{n}(T_{\mu^{\prime }}^{n}\left(P_{\hat{X}|X}\left(\cdot |x^{n}\right)|x^{n}\right)\geq 1-\mu^{\prime }$ for *n* sufficiently large.

Combining (A23) and (A27), we obtain

(A28)H(X^n|Xn)≥n(1−μ′)2H(X^|X)∑xn∈Tμn(PX)PXn(xn)(A29)≥n(1−μ′)2(1−μ)H(X^|X),

where (A29) follows because the AEP [36] (Theorem 3.1.1) implies that $P_{X}^{n}\left(T_{\mu }^{n}\left(P_{X}\right)\right)\geq 1-\mu$ for *n* sufficiently large.

Hence, we have

(A30)I(Xn;X^n)=H(X^n)−H(X^n|Xn)(A31)≤nH(X^)−H(X^n|Xn)(A32)≤nH(X^)−n(1−μ′′)H(X^|X)(A33)=nI(X;X^)+nμ′′H(X^|X)(A34)≤nL+nμ′′·log|X^|(A35)=nL+nζ,

where $\mu^{\prime \prime }≜1-{\left(1-\mu^{\prime }\right)}^{2}\left(1-\mu \right)\geq 0$, and $\zeta ≜\mu^{\prime \prime }\cdot \log\left|\hat{X}\right|$.

Next, consider the privacy analysis under H=1. Please note that when $P_{X}=Q_{X}$, the analysis is similar to that of H=0. Thus, we assume that $P_{X}\neq Q_{X}$ in the following. From (A22), the privacy measure $T_{n}$ satisfies:

(A36) $$nT_{n}=I\left(X^{n};{\hat{X}}^{n}\right)\leq H\left({\hat{X}}^{n}\right).$$

To upper bound $H({\hat{X}}^{n})$, we calculate the probability $P_{{\hat{X}}^{n}}\left({\hat{x}}^{n}\right)$ for ${\hat{x}}^{n}=0^{n}$ as follows:

(A37)PX^n(0n)=∑xn∈Tμn(PX)PX^|Xn(0n|xn)·QXn(xn)+∑xn∉Tμn(PX)PX^|Xn(0n|xn)·QXn(xn)(A38)=∑xn∉Tμn(PX)PX^|Xn(0n|xn)·QXn(xn)(A39)=∑xn∉Tμn(PX)QXn(xn)(A40)=1−QXnTμn(PX)(A41)≥1−2−n(D(PX∥QX)+δ(μ))≜1−γn,

where $\gamma_{n}\to 0$ exponentially fast as n→∞. Here, (A38) follows because if $x^{n}\in T_{\mu }^{n}\left(P_{X}\right)$, then $P_{\hat{X}|X}^{n}\left(0^{n}|x^{n}\right)=0$, (A39) follows because when $x^{n}\notin T_{\mu }^{n}\left(P_{X}\right)$, then $P_{\hat{X}|X}^{n}\left(0^{n}|x^{n}\right)=1$ and (A41) follows from Sanov’s theorem and the continuity of the relative entropy in its first argument [38] (Lemma 1.2.7).

Write $H({\hat{X}}^{n})$ as $H(P_{{\hat{X}}^{n}})$ and let $P_{0^{n}}$ be the distribution on ${\hat{X}}^{n}$ that places all its probability mass on $0^{n}\in {\hat{X}}^{n}$. Since $H(P_{0^{n}})=0$, by the uniform continuity of entropy [38] (Lemma 1.2.7),

(A42) $$\begin{array}{c} H\left(P_{{\hat{X}}^{n}}\right)\leq 2|P_{{\hat{X}}^{n}}-P_{0^{n}}|\cdot \log\frac{|\hat{X}{|}^{n}}{2|P_{{\hat{X}}^{n}}-P_{0^{n}}|}. \end{array}$$

Since $\gamma_{n}\to 0$ exponentially fast, the same holds true for $|P_{{\hat{X}}^{n}}-P_{0^{n}}|$ and so by (A42), $H\left(P_{{\hat{X}}^{n}}\right)=H\left({\hat{X}}^{n}\right)\to 0$. Therefore, under H=1, we have $T_{n}\to 0$ as n→∞.

Letting n→∞ and then letting $\mu ,\mu^{\prime },\gamma \to 0$, we obtain ${\tilde{\theta }}_{\mu }\to \theta$ and ${lim sup}_{n\to n}T_{n}\leq L$, with θ given by the RHS of (11). This establishes the proof of Theorem 1.

## Appendix B. Proof of Theorem 2

*Achievability*: The analysis is based on the scheme of Section 4.2. It follows similar steps as in [1]. Recall the definition of the event E in (A4). Consider the type-I error probability as follows:

(A43)αn≤PrM=0orE|H=0(A44)≤PrM=0|H=0+PrE|M≠0,H=0(A45)≤ϵ/2+ϵ/2(A46)=ϵ,

where (A46) follows from covering lemma [34] (Lemma 3.3) and the rate constraint in (15), and also the Markov lemma [34] (Lemma 12.1). Now, consider the type-II error probability as follows:

(A47)βn=Pr[H^=0|H=1](A48)=Pr[H^=0,M≠0|H=1](A49)≤Pr[H^=0|H=1,M≠0](A50)=Pr[H^=0|H=1,M=1],

where the last equality follows from the symmetry of the code construction. Now, the average of type-II error probability over all codebooks satisfies:

(A51) $$\begin{array}{c} E_{C}\left[\beta_{n}\right]\leq 2^{-n[I(U;Y)-\delta (\mu )]}, \end{array}$$

where δ(μ) is a function that tends to zero as μ→0. The privacy analysis is straightforward since the privacy mechanism is memoryless whence we have

(A52) $$\begin{array}{c} \frac{1}{n}I\left(X^{n};{\hat{X}}^{n}\right)=I\left(X;\hat{X}\right)=L+\zeta , \end{array}$$

where the last equality follows from the privacy constraint in (15). This concludes the proof of achievability.

*Converse*: Now, we prove the strong converse. It involves an extension of the η-image characterization technique [4,38]. The proof steps are given as follows. First, we find a truncated distribution $P_{{\underline{\hat{X}}}^{n}}$ which is arbitrarily close to $P_{\hat{X}}^{n}$ in terms of entropy. Then, we analyze the type-II error probability under a constrained type-I error probability. Finally, a single-letter characterization of the rate and privacy constraints is given.

(1) *Construction of a Truncated Distribution:*

Since the privacy machanism is memoryless, we conclude that $\left(X^{n},{\hat{X}}^{n},Y^{n}\right)$ is i.i.d. according to $P_{X\hat{X}Y}≜P_{\hat{X}|X}P_{XY}$. For a given $P_{\hat{X}Y}$, define $V^{n}\left(y^{n}|{\hat{x}}^{n}\right)≜P_{Y|\hat{X}}^{n}\left(y^{n}|{\hat{x}}^{n}\right)$ for all ${\hat{x}}^{n}\in {\hat{X}}^{n}$ and $y^{n}\in Y^{n}$. A set $B\subseteq Y^{n}$ is an η-image of the set $A\subseteq {\hat{X}}^{n}$ over the channel $V^{n}$ if

(A53) $$V^{n}\left(B|{\hat{x}}^{n}\right)\geq \eta ,\;\forall {\hat{x}}^{n}\in A.$$

The privacy mechanism is the same under both hypotheses, thus, we can define the acceptance region based on $\left({\hat{x}}^{n},y^{n}\right)$ as follows:

(A54) $$\begin{array}{c} A_{n}≜\left\{\left({\hat{x}}^{n},y^{n}\right):g^{\left(n\right)}\left(y^{n},\phi^{\left(n\right)}\left({\hat{x}}^{n}\right)\right)=0\right\}. \end{array}$$

For any encoding function $\phi^{\left(n\right)}$ and an acceptance region $A_{n}\subseteq {\hat{X}}^{n}\times Y^{n}$, let $\tau_{n}$ denote the cardinality of codebook and define the following sets:

(A55) $$\begin{array}{cc} C_{i} & \overset{\Delta }{=}\left\{{\hat{x}}^{n}\in {\hat{X}}^{n}:\phi^{\left(n\right)}\left({\hat{x}}^{n}\right)=i\right\}, \end{array}$$

(A56) $$\begin{array}{cc} \;D_{i} & \overset{\Delta }{=}\left\{y^{n}\in Y^{n}:g^{\left(n\right)}\left(y^{n},i\right)=0\right\},\;1\leq i\leq \tau_{n}. \end{array}$$

The acceptance region can be written as follows:

(A57) $$\begin{array}{c} A_{n}=\overset{\tau_{n}}{\underset{i=1}{⋃}}\left(C_{i}\times D_{i}\right), \end{array}$$

where $C_{i}\cap C_{j}=\phi$ for all i≠j. Define the set $B_{n}\left(\eta \right)$ as follows:

(A58) $$\begin{array}{c} B_{n}\left(\eta \right)≜\left\{{\hat{x}}^{n}:V^{n}\left(D_{\phi^{\left(n\right)}\left({\hat{x}}^{n}\right)}|{\hat{x}}^{n}\right)\geq \eta \right\}. \end{array}$$

Fix ϵ∈[0,1) and notice that the type-I error probability is upper-bounded as

(A59) $$\begin{array}{cc} \alpha_{n} & =P_{\hat{X}Y}^{n}\left(A_{n}^{c}\right)\leq \epsilon , \end{array}$$

which we can write equivalently as

(A60)1−ϵ≤PX^YnAn(A61)=∑x^n∈Bn(η)PX^n(x^n)VnDϕ(n)(x^n)|x^n+∑x^n∈Bnc(η)PX^n(x^n)VnDϕ(n)(x^n)|x^n(A62)≤PX^nBn(η)+η1−PX^n(Bn(η)),

where the first term is because $V^{n}\left(D_{\phi^{\left(n\right)}\left({\hat{x}}^{n}\right)}|{\hat{x}}^{n}\right)\leq 1$; and the second term is because for any ${\hat{x}}^{n}\in B_{n}^{c}\left(\eta \right)$, we have $V^{n}\left(D_{\phi^{\left(n\right)}\left({\hat{x}}^{n}\right)}|{\hat{x}}^{n}\right)<\eta$.

In what follows, let $\eta =\frac{1-\epsilon }{2}$. Inequality (A62) implies

(A63) $$\begin{array}{c} P_{\hat{X}}^{n}\left(B_{n}\left(\eta \right)\right)\geq \frac{1-\epsilon }{1+\epsilon }. \end{array}$$

Let $\mu_{n}=n^{-1/3}$. For the typical set $T_{\mu_{n}}^{n}\left(P_{\hat{X}}\right)$, we have

(A64) $$\begin{array}{c} P_{\hat{X}}^{n}\left(T_{\mu_{n}}^{n}\left(P_{\hat{X}}\right)\right)\geq 1-\frac{|\hat{X}|}{2\mu_{n}n}. \end{array}$$

Hence,

(A65)PX^nTμnn(PX^)∩Bn(η)≥PX^nTμnn(PX^)+PX^nBn(η)−1(A66)≥1−ϵ1+ϵ−|X^|2μnn.

For any $0<\delta <\frac{1-\epsilon }{1+\epsilon }$ and for sufficiently large *n*,

(A67) $$\begin{array}{cc} P_{\hat{X}}^{n}\left(T_{\mu_{n}}^{n}\left(P_{\hat{X}}\right)\cap B_{n}\left(\eta \right)\right) & \geq \delta . \end{array}$$

We can also write $T_{\mu_{n}}^{n}\left(P_{\hat{X}}\right)$ as

(A68) $$\begin{array}{c} T_{\mu_{n}}^{n}\left(P_{\hat{X}}\right)=\underset{{\hat{P}}_{\hat{X}}:\left|{\hat{P}}_{\hat{X}}-P_{\hat{X}}\right|\leq \mu_{n}}{⋃}T^{n}\left({\hat{P}}_{\hat{X}}\right). \end{array}$$

Combining the above equations, we get

(A69) $$\begin{array}{c} \sum_{{\hat{P}}_{\hat{X}}:\left|{\hat{P}}_{\hat{X}}-P_{\hat{X}}\right|\leq \mu_{n}}\;\;\;\;P_{\hat{X}}^{n}\left(T^{n}\left({\hat{P}}_{\hat{X}}\right)\cap B_{n}\left(\eta \right)\right)\geq \delta . \end{array}$$

Let ${\tilde{P}}_{\hat{X}}$ denote the type which maximizes the $P_{\hat{X}}^{n}$-probability of the type class among all such types. As there exist at most ${\left(n+1\right)}^{|\hat{X}|}$ possible types, it holds that

(A70) $$\begin{array}{c} P_{\hat{X}}^{n}\left(T^{n}\left({\tilde{P}}_{\hat{X}}\right)\cap B_{n}\left(\eta \right)\right)\geq \frac{\delta }{{\left(n+1\right)}^{|\hat{X}|}}. \end{array}$$

Define the set $\Psi_{n}\left(\eta \right)≜T^{n}\left({\tilde{P}}_{\hat{X}}\right)\cap B_{n}\left(\eta \right)$. We can write the probability in (A70) as

(A71)PX^nTn(P˜X^)∩Bn(η)=∑x^n∈Ψn(η)PX^n(x^n)(A72)=∑x^n∈Ψn(η)2−nD(P˜X^∥PX^)+HP˜X^(X^)(A73)≤∑x^n∈Ψn(η)2−n[H(X^)−δ1]

where $\delta_{1}\to 0$ as n→∞ due to the fact that $D({\tilde{P}}_{\hat{X}}∥P_{\hat{X}})\geq 0$ and $|{\tilde{P}}_{\hat{X}}-P_{\hat{X}}|\leq \mu_{n}$ so the entropies are also arbitrarily close. It then follows from (A70) and (A73) that

(A74) $$\begin{array}{c} \frac{1}{n}\log\left|\Psi_{n}\left(\eta \right)\right|\geq H\left(\hat{X}\right)-\delta_{2}, \end{array}$$

where $\delta_{2}\to 0$ as $\mu_{n}\to 0$.

The encoding function $\phi^{\left(n\right)}$ partitions the set $\Psi_{n}\left(\eta \right)$ into $\tau_{n}$ non-intersecting subsets ${\left\{S_{i}\right\}}_{i=1}^{\tau_{n}}$ such that $\phi^{\left(n\right)}\left({\hat{x}}^{n}\right)=i$ for any ${\hat{x}}^{n}\in S_{i}$. Define the following distribution:

(A75) $$\begin{array}{cc} \;\;P_{{\underline{\hat{X}}}^{n}}\left({\hat{x}}^{n}\right) & ≜\;\frac{P_{\hat{X}}^{n}\left({\hat{x}}^{n}\right)\cdot 1\;\left\{{\hat{x}}^{n}\in \Psi_{n}\left(\eta \right)\right\}}{P_{\hat{X}}^{n}\left(\Psi_{n}\left(\eta \right)\right)}. \end{array}$$

Please note that this distribution, henceforth denoted as $P^{\left(n\right)}$, corresponds to a uniform distribution over $\Psi_{n}\left(\eta \right)$ because all sequences in $\Psi_{n}\left(\eta \right)$ have the same type ${\tilde{P}}_{\hat{X}}$, and the probability is uniform on a type class under any i.i.d. measure.

Finally, define the following truncated joint distribution:

(A76) $$\begin{array}{c} P_{\underline{M}{\underline{X}}^{n}{\underline{\hat{X}}}^{n}{\underline{Y}}^{n}}\left(m,x^{n},{\hat{x}}^{n},y^{n}\right)≜1\left\{\phi^{\left(n\right)}\left(x^{n}\right)=m\right\}P_{X|\hat{X}}^{n}\left(x^{n}|{\hat{x}}^{n}\right)P_{{\underline{\hat{X}}}^{n}}\left({\hat{x}}^{n}\right)P_{Y|X}^{n}\left(y^{n}|x^{n}\right). \end{array}$$

(2) *Analysis of Type-II Error Exponent:*

The proof of the upper bound on the error exponent relies on the following Lemma A1. For a set $A\subseteq {\hat{X}}^{n}$, let B(A,η) denote the collection of all η-images of *A*, define $Q_{X\hat{X}Y}≜P_{\hat{X}|X}Q_{XY}$ and

(A77) $$\begin{array}{c} \kappa_{V^{n}}\left(A,Q_{\hat{X}Y},\eta \right)≜\frac{\min_{B\in B(A,\eta )}Q_{\hat{X}Y}^{n}\left(A\times B\right)}{P_{\hat{X}}^{n}\left(A\right)}. \end{array}$$

This quantity is a generalization of the minimum cardinality of the η-images in [38] and is closely related to the minimum type-II error probability associated with the set *A*.

For the testing against independence setup, $Q_{\hat{X}Y}=P_{\hat{X}}\cdot P_{Y}$, and thus

(A78) $$\begin{array}{c} \frac{Q_{\hat{X}Y}^{n}\left(A\times B\right)}{P_{\hat{X}}^{n}\left(A\right)}=\frac{P_{\hat{X}}^{n}\left(A\right)P_{Y}^{n}\left(B\right)}{P_{\hat{X}}^{n}\left(A\right)}=P_{Y}^{n}\left(B\right), \end{array}$$

and $\kappa_{V^{n}}\left(A,Q_{\hat{X}Y},\eta \right)$ is simply written as $\kappa_{V^{n}}\left(A,\eta \right)$ and is given by

(A79) $$\begin{array}{c} \kappa_{V^{n}}\left(A,\eta \right)≜\min_{B\in B(A,\eta )}P_{Y}^{n}\left(B\right). \end{array}$$

**Lemma** **A1** (Lemma 3 in [4])**.** *For any set $A\subseteq {\hat{X}}^{n}$, consider a distribution $P_{A}^{\left(n\right)}$ over A and let $P_{A}^{\left(n\right)}V^{n}$ be its corresponding output distribution induced by the channel $V^{n}$, i.e.,*

(A80) $$P_{A}^{\left(n\right)}V^{n}\left(y^{n}\right)≜\sum_{{\hat{x}}^{n}\in A}P_{A}^{\left(n\right)}\left({\hat{x}}^{n}\right)V^{n}\left(y^{n}|{\hat{x}}^{n}\right).$$

*Then, for every $\delta^{\prime }>0$, 0<η<1, we have*

(A81) $$\begin{array}{c} \kappa_{V^{n}}\left(A,\eta \right)\geq 2^{-D\left(P_{A}^{\left(n\right)}V^{n}∥P_{Y}^{n}\right)-n\delta^{\prime }} \end{array}$$

*for sufficiently large n.*

Let $P_{i}^{\left(n\right)}V^{n}$ be the distribution of the random variable ${\underline{Y}}^{n}$ given $\underline{M}=i$. The type-II error probability can be lower-bounded as:

(A82)βn≥∑x^n∈Ψn(η)PX^nx^n·PYnDϕ(n)(x^n)(A83)=∑i=1τnPX^n(Si)·PYn(Di)(A84)≥∑i=1τnPX^n(Si)·κVn(Si,η)(A85)=PX^nΨn(η)·∑i=1τnP(n)(Si)·κVn(Si,η)(A86)≥2−nδ′·PX^nΨn(η)·∑i=1τnP(n)(Si)·2−DPi(n)Vn∥PYn(A87)≥2−nδ′·PX^nΨn(η)·2−∑i=1τnP(n)(Si)·DPi(n)Vn∥PYn(A88)≥2−nδ′δ(n+1)|X^|·2−∑i=1τnP(n)(Si)·DPi(n)Vn∥PYn,

where (A84) follows from the definition of $\kappa_{V^{n}}\left(S_{i},\eta \right)$, (A86) follows because Lemma A1 implies that for any distribution $P_{i}^{\left(n\right)}$ over the set $S_{i}$ it holds that $\kappa_{V^{n}}\left(S_{i},\eta \right)\geq 2^{-D\left(P_{i}^{\left(n\right)}V^{n}∥P_{Y}^{n}\right)-n\delta^{\prime }}$, (A87) follows because of the convexity of the function $t\mapsto 2^{t}$, and (A88) follows by (A70) and the fact that Pr(A)≥Pr(A∩B). Hence,

(A89) $$\begin{array}{c} \;\;\;-\frac{1}{n}\log\beta_{n}-\delta^{\prime \prime }\leq \frac{1}{n}\sum_{i=1}^{\tau_{n}}P^{\left(n\right)}\left(S_{i}\right)\cdot D\left(P_{i}^{\left(n\right)}V^{n}∥P_{Y}^{n}\right),\;\; \end{array}$$

where $\delta^{\prime \prime }≜\delta^{\prime }-\frac{1}{n}\log\frac{\delta }{{\left(n+1\right)}^{|\hat{X}|}}$.

(3) *Single-letterization Steps and Analyses of Rate and Privacy Constraints:*

In the following, we proceed to provide a single-letter characterization of the upper bound in (A89). Considering the fact that $P^{\left(n\right)}\left(S_{i}\right)=P_{\underline{M}}\left(i\right)$, the right-hand-side of (A89) can be upper-bounded as follows:

(A90)1n∑i=1τnP(n)(Si)·D(Pi(n)Vn∥PYn)=1n∑i=1τn∑yn∈YnPM_Y_n(i,yn)logPY_n|M_(yn|i)PYn(yn)(A91)=−1nH(Y_n|M_)−1n∑yn∈YnPY_n(yn)logPYn(yn)(A92)=−1nH(Y_n|M_)−1n∑yn∈YnPY_n(yn)∑t=1nlogPY(yt)(A93)=−1nH(Y_n|M_)−1n∑t=1n∑yn∈YnPY_n(yn)logPY(yt)(A94)=−1nH(Y_n|M_)−1n∑t=1n∑yt∈YPY_t(yt)logPY(yt)(A95)=−1nH(Y_n|M_)+1n∑t=1nH(Y_t)+D(PY_t∥PY)(A96)=1n∑t=1nH(Y_t)−H(Y_t|M_,Y_t−1)+D(PY_t∥PY)(A97)≤1n∑t=1nI(M_,X_t−1,X^_t−1;Y_t)+1n∑t=1nD(PY_t∥PY)(A98)=1n∑t=1nI(U_t;Y_t)+1n∑t=1nD(PY_t∥PY)(A99)=I(U_;Y_)+D(PY_∥PY).

Here, (A96)–(A99) are justified in the following:

- (A96) follows by the chain rule;
- (A97) follows from the Markov chain ${\underline{Y}}^{t-1}\to \left(\underline{M},{\underline{X}}^{t-1},{\underline{\hat{X}}}^{t-1}\right)\to {\underline{Y}}_{t}$;
- (A98) follows from the definition
  (A100) $$\begin{array}{c} {\underline{U}}_{t}≜\left(\underline{M},{\underline{X}}^{t-1},{\underline{\hat{X}}}^{t-1}\right); \end{array}$$
- (A99) follows by defining a time-sharing random variable *T* over {1,…,n} and the following
  (A101) $$\begin{array}{c} \underline{U}\overset{\Delta }{=}\left({\underline{U}}_{T},T\right),\;\underline{Y}\overset{\Delta }{=}{\underline{Y}}_{T}. \end{array}$$

This leads to the following upper-bound on the type-II error exponent:

(A102) $$\begin{array}{c} -\frac{1}{n}\log\beta_{n}\leq I\left(\underline{U};\underline{Y}\right)+D\left(P_{\underline{Y}}∥P_{Y}\right)+\delta^{\prime \prime }. \end{array}$$

Next, the rate constraint satisfies the following:

(A103)nR≥H(M_)(A104)≥I(M_;X^_n)(A105)=H(X^_n)−H(X^_n|M_)(A106)=log|Ψn(η)|−H(X^_n|M_)(A107)≥n(H(X^)−δ2)−H(X^_n|M_)(A108)=nH(X^)−∑t=1nH(X^_t|X^_t−1,M_)−nδ2(A109)=nH(X^)−∑t=1nH(X^_t|U_t)−nδ2(A110)=nH(X^)−nH(X^_|U_)−nδ2

where (A106) follows because the distribution $P_{{\underline{\hat{X}}}^{n}}$ is uniform over the set $\Psi_{n}\left(\eta \right)$; (A107) follows from (A74); (A109) follows from the definition in (A100); (A110) follows by defining $\underline{\hat{X}}≜{\underline{\hat{X}}}_{T}$.

Finally, the privacy measure satisfies the following:

(A111)nL≥I(Xn;X^n)(A112)≥I(X_n;X^_n)(A113)=H(X^_n)−H(X^_n|X_n)(A114)=log|Ψn(η)|−H(X^_n|X_n)(A115)=nH(X^)−δ2−H(X^_n|X_n)(A116)=nH(X^)−δ2−∑t=1nH(X^_t|X^_t−1,X_n)(A117)≥nH(X^)−δ2−∑t=1nH(X^_t|X_t)(A118)=nH(X^)−nH(X^_|X_)−nδ2,

where (A112) follows because $\left({\underline{X}}^{n},{\underline{\hat{X}}}^{n}\right)$ are functions of $\left(X^{n},{\hat{X}}^{n}\right)$ and from data processing inequality, (A114) follows because $P_{{\underline{\hat{X}}}^{n}}$ is uniform over the set $\Psi_{n}\left(\eta \right)$ (see definition in (A75)), (A115) follows from (A74), and (A118) follows by defining $\underline{X}≜\left({\underline{X}}_{T},T\right)$, $\underline{\hat{X}}≜\left({\underline{\hat{X}}}_{T},T\right)$ and choosing *T* uniformly over {1,…,n}.

Since $\Psi_{n}\left(\eta \right)\subseteq T^{n}\left({\tilde{P}}_{\hat{X}}\right)$, for any $\hat{x}\in \hat{X}$,

(A119)PX^_(x^)=1n∑t=1nPX^_t(x^)(A120)=∑x^n∈Ψn(η)Nx^|x^nn·|Ψn(η)|(A121)=P˜X^(x^).

Recall that $|{\tilde{P}}_{\hat{X}}-P_{\hat{X}}|\leq \mu_{n}$ with $\mu_{n}=n^{-1/3}$. Hence, from (A121), it holds that $|P_{\underline{\hat{X}}}-P_{\hat{X}}|\leq \mu_{n}$. By the definitions of $\underline{\hat{X}}$, $\underline{X}$ and $\underline{Y}$, we have $P_{\hat{X}|X}=P_{\underline{\hat{X}}|\underline{X}}$ and $P_{Y|X}=P_{\underline{Y}|\underline{X}}=V$. The random variable *U* is chosen over the same alphabet as $\underline{U}$ and such that $P_{U|\hat{X}}=P_{\underline{U}|\underline{\hat{X}}}$.

Since $P_{Y}\left(y\right)>0$ for all y∈Y, letting n→∞ and $\mu_{n}\to 0$ and the uniform continuity of the involved information-theoretic quantities yields the following upper bound on the optimal error exponent:

(A122) $$\begin{array}{c} \theta_{\epsilon }^{*}\left(R,L\right)\leq I\left(U;Y\right), \end{array}$$

subject to the rate constraint:

(A123) $$\begin{array}{c} R\geq I(U;\hat{X}), \end{array}$$

and the privacy constraint:

(A124) $$\begin{array}{ccc} L & \geq & I(X;\hat{X}). \end{array}$$

This concludes the proof of converse.

## Appendix C. Proof of Converse of Proposition 1

We simplify Theorem 2 for the proposed binary setup. As discussed in Section 4.3, from the fact that $|\hat{X}|=2$ and the symmetry of the source *X* on its alphabet, without loss of optimality, we can choose $P_{\hat{X}|X}$ to be a BSC. First, consider the rate constraint:

(A125)R≥I(U;X^)(A126)=H(X^)−H(X^|U)(A127)=1−H(X^|U),

which can be equivalently written as the following:

(A128) $$\begin{array}{cc} H(\hat{X}|U) & \geq 1-R. \end{array}$$

Also, the privacy criterion can be simplified as follows:

(A129)L≥I(X^;X)(A130)=H(X^)−H(X^|X)(A131)=1−H(X^|X)(A132)=1−H(Z^),

which can be equivalently written as

(A133) $$\begin{array}{c} H(\hat{Z})\geq 1-L. \end{array}$$

Now, consider the error exponent θ as follows:

(A134)θ≤I(U;Y)(A135)=H(Y)−H(Y|U)(A136)=H(Y)−H(X⊕N|U)(A137)=H(Y)−H(X^⊕Z^⊕N|U)(A138)≤H(Y)−hbhb−1(H(X^|U))⋆hb−1(1−L)⋆q(A139)≤H(Y)−hbhb−1(1−R)⋆hb−1(1−L)⋆q,

where (A138) follows from Mrs. Gerber’s lemma [39] (Theorem 1) and the fact that $\left(\hat{Z},N\right)$ is independent of *U* and also from (A133); (A139) follows from (A128). This concludes the proof of the proposition.

## Appendix D. Euclidean Approximation of Testing agianst Independence

We analyze the Euclidean approximation with the parameters, W, $\psi_{u}\left(\hat{x}\right)$, $\phi_{\hat{x}}\left(x\right)$ and $\Lambda_{u}\left(x\right)$ defined in Section 4.4. Notice that since $U\to \hat{X}\to X\to Y$ forms a Markov chain, it holds that, for any u∈U,

(A140) $$\begin{array}{c} P_{Y|U=u}=WP_{X|U=u}. \end{array}$$

Now, consider the following chain of equalities for any x∈X:

(A141)PX|U(x|u)=∑x^∈X^PXX^|U(x,x^|u)(A142)=∑x^∈X^PX^|U(x^|u)PX|X^,U(x|x^,u)(A143)=∑x^∈X^PX^|U(x^|u)PX|X^(x|x^)(A144)=∑x^∈X^PX^(x^)+ψu(x^)PX(x)+ϕx^(x)(A145)=PX(x)+∑x^∈X^ψu(x^)ϕx^(x)+∑x^∈X^PX^(x^)ϕx^(x)+PX(x)∑x^∈X^ψu(x^)(A146)=PX(x)+∑x^∈X^ψu(x^)ϕx^(x),

where (A143)—(A146) are justified in the following:

- (A143) follows from the Markov chain $U\to \hat{X}\to X$ where given $\hat{X}$, *U* and *X* are independent;
- (A144) follows from (30) and (36);
- (A146) follows from (31) and also from (36) which yields the following:
  (A147) $$\begin{array}{c} \sum_{\hat{x}\in \hat{X}}P_{\hat{X}}\left(\hat{x}\right)\cdot \phi_{\hat{x}}\left(x\right)=0. \end{array}$$

With the definition of $\Lambda_{u}\left(x\right)$ in (42), we can write

(A148) $$\begin{array}{cc} P_{X|U}\left(x|u\right) & =P_{X}\left(x\right)+\Lambda_{u}\left(x\right),\;\forall x\in X,u\in U. \end{array}$$

Thus, we get

(A149)PY|U=u=WPX+WΛu(A150)=PY+WΛu.

Applying the $\chi^{2}$-approximation and using (A150), we can rewrite I(U;Y) as follows:

(A151) $$\begin{array}{cc} I(U;Y) & \approx \frac{1}{2}\;\log e\;\sum_{u\in U}P_{U}\left(u\right)\;{∥{\left[\sqrt{P_{Y}}\right]}^{-1}W\Lambda_{u}∥}^{2} \end{array}$$

The above approximation with the definition of the vector $\Lambda_{u}$ in (43) yields the optimization problem in (45).

## Appendix E. Proof of Proposition 2

*Achievability*: We specialize the achievable scheme of Theorem 2 to the proposed Gaussian setup. We choose the auxiliary random variables as in (63) and (65). Notice that from the Markov chain $U\to \hat{X}\to X\to Y$ and also the Gaussian choice of $\hat{X}$ in (63) which was discussed in Section 4.5, we can write $Y=\rho \hat{X}+F$ where $F∼N\left(0,1-\rho^{2}\cdot \left(1-2^{-2L}\right)\right)$ is independent of $\hat{X}$. These choices of auxiliary random variables lead to the following rate constraint:

(A152) $$\begin{array}{c} R\geq \frac{1}{2}\log\left(\frac{1-2^{-2L}}{\beta^{2}}\right), \end{array}$$

which can be equivalently written as:

(A153) $$\begin{array}{c} 2^{-2R}\cdot \left(1-2^{-2L}\right)\leq \beta^{2}. \end{array}$$

The optimal error exponent is also lower bounded as follows

(A154) $$\begin{array}{c} \theta_{\epsilon }^{*}\left(R,L\right)\geq \frac{1}{2}\log\left(\frac{1}{1-\rho^{2}\cdot \left(1-2^{-2L}-\beta^{2}\right)}\right). \end{array}$$

Combining (A153) and (A154) gives the lower bound on the error exponent in (64).

*Converse*: Consider the following upper bound on the optimal error exponent in Theorem 2:

(A155)θϵ*(R,L)≤I(U;Y)(A156)=h(Y)−h(Y|U)(A157)=12log2πe−h(Y|U)(A158)=12log2πe−hρX^+F|U(A159)≤12log2πe−12log22hρX^|U+2πe1−ρ2·(1−2−2L)(A160)≤12log2πe−12logρ222hX^|U+2πe1−ρ2·(1−2−2L),

where (A159) follows from the entropy power inequality (EPI) [34] (Chapter 2). Now, consider the rate constraint as follows:

(A161)R≥I(X^;U)(A162)=h(X^)−h(X^|U)(A163)=12log2πe1−2−2L−h(X^|U),

which is equivalent to

(A164) $$\begin{array}{c} 2^{2h(\hat{X}|U)}\geq 2\pi e\cdot 2^{-2R}\cdot \left(1-2^{-2L}\right). \end{array}$$

Considering (A160) with (A164) yields the following upper bound on the error exponent:

(A165)θϵ*(R,L)≤12log2πe−12log2πeρ22−2R1−2−2L+2πe1−ρ2(1−2−2L)(A166)=12log11−ρ2(1−2−2R)(1−2−2L).

This concludes the proof of the proposition.
